# Disproportionality Analysis and Timing of Drug-Associated Guillain–Barré Syndrome Onset Based on the Japanese Adverse Drug Event Report Database

**DOI:** 10.3390/ph19050688

**Published:** 2026-04-28

**Authors:** Shinya Toriumi, Yousuke Kurihara, Komei Shimokawa, Arihito Tanaka, Norito Araki, Osamu Kawai, Yasoo Sugiura, Yoshihiro Uesawa

**Affiliations:** 1 Department of Medical Molecular Informatics, Meiji Pharmaceutical University, Kiyose 204-8588, Tokyo, Japan; 2 Department of Pharmacy, National Hospital Organization Kanagawa Hospital, Hadano 257-8585, Kanagawa, Japanshimokawa.komei.gr@mail.hosp.go.jp (K.S.); 3 Department of Respiratory Medicine, National Hospital Organization Kanagawa Hospital, Hadano 257-8585, Kanagawa, Japan; 4 Department of General Thoracic Surgery, National Hospital Organization Saitama Hospital, Wako 351-0102, Saitama, Japan

**Keywords:** Guillain–Barré syndrome, acute inflammatory demyelinating polyneuropathy, Japanese adverse drug event report database, pharmacovigilance, spontaneous reporting system, disproportionality analysis, time-to-onset analysis, Weibull distribution, vaccines, immune checkpoint inhibitors

## Abstract

**Background**: Guillain–Barré syndrome (GBS) is an autoimmune peripheral neuropathy that can lead to paralysis and respiratory failure. In addition to infections, several drugs have been suggested as potential triggers of GBS. This study investigated drug-associated GBS using a spontaneous adverse event reporting database through disproportionality analysis for signal detection and time-to-onset analysis. **Methods**: The Japanese Adverse Drug Event Report (JADER) database was analyzed to assess more than 4000 drugs for potential associations with GBS. Signal detection was performed using reporting odds ratios, Fisher’s exact test, and total report counts. For vaccines and immune checkpoint inhibitors, time-to-onset patterns were further evaluated using Weibull distribution analysis. **Results**: Disproportionality signals suggesting potential associations with GBS were identified for 45 drugs, including vaccines, immune checkpoint inhibitors, tumor necrosis factor-α inhibitors, other anticancer drugs, antifungal agents, and interferons. Reports following vaccination were most frequently observed within 1–3 weeks after administration of coronavirus disease 2019 (COVID-19), influenza, and pneumococcal vaccines, and within 1–3 months after human papillomavirus 2-valent vaccination, with a gradual decrease thereafter. Reports following immune checkpoint inhibitor use were most frequently observed 1–3 months after nivolumab, ipilimumab, and pembrolizumab administration, whereas atezolizumab showed a peak in reporting within 1–3 weeks. In contrast to vaccine-related reports, no clear temporal trend in reporting was observed. **Conclusions**: Drugs that modulate immune function, including vaccines and immune checkpoint inhibitors, may be associated with reported GBS events. Vaccine-related reports showed an early concentration in time to onset, whereas immune checkpoint inhibitor-related reports did not demonstrate a clear temporal pattern. These findings should be interpreted as hypothesis-generating and warrant further investigation.

## 1. Introduction

Guillain–Barré syndrome (GBS) is an autoimmune peripheral neuropathy that develops after infections, such as diarrhea or the common cold [1]. GBS progresses over a period of days to weeks, with severe cases leading to paralysis and respiratory failure [2,3]. The pathogenesis of GBS has been attributed to cross-reactive antibodies induced by infection that target components of the neuronal membrane, resulting in demyelination [4,5]. The incidence of GBS is 1.12 cases per 100,000 people per year, although it varies by region and shows a slight male predominance [6]. Although treatments for GBS include intravenous immunoglobulin and plasma exchange, early diagnosis and treatment are crucial [7]. GBS is often triggered by infection; however, reports of drug-associated GBS, including reports following vaccination [8] and immune checkpoint inhibitor therapy [9], have also been described. Because drug-associated GBS is rare, data to support pharmaceutical management and adverse event monitoring remain limited.

Spontaneous reporting systems, which collect post-marketing adverse event reports from patients, healthcare professionals, pharmaceutical companies, and other sources, play an important role in epidemiological research and drug safety assessment, despite inherent limitations in establishing causality and various reporting biases [10,11,12]. These systems cover large populations, including children, older adults, and patients with renal or hepatic impairment, and they contain large numbers of reports that reflect prescribing patterns and real-world conditions of use. Accordingly, spontaneous adverse event reporting databases have been widely used to detect signals of potential associations between various drugs and adverse events [13,14,15]. The Japanese Adverse Drug Event Report (JADER) database, established by the Pharmaceuticals and Medical Devices Agency (PMDA), is a spontaneous reporting database containing approximately 1,500,000 adverse drug reaction records [16]. In spontaneous reporting system databases, such as JADER, disproportionality-based methods, including reporting odds ratio (ROR) analysis, can be used to explore potential drug–event associations in an exploratory, hypothesis-generating manner. Because JADER includes information on the interval between drug administration and adverse event onset, temporal analysis is also possible [17]. JADER-based evaluations can therefore provide useful information for adverse event management and drug safety.

The present study aimed to detect disproportionality signals of potential associations between drugs and GBS and to evaluate the time to onset and related reporting characteristics.

## 2. Results

### 2.1. JADER Analysis Dataset

**Figure 1:**
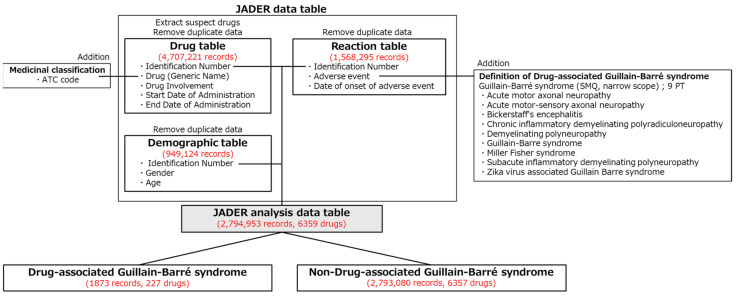
Procedure for creating the analysis data table from JADER. Duplicate data were removed from the Drug, Reaction, and Demographic tables. Only “suspected drugs” were extracted from the Drug table. ATC codes for therapeutic classification were added to the Drug table. Drug-associated GBS was defined using the Guillain–Barré syndrome Standardized MedDRA Query (SMQ) among the adverse events in the Reaction table. Furthermore, the Drug, Reaction, and Demographic tables were combined using identification numbers. Of the 2,794,953 reports included in the JADER analysis data table, 1873 involved drug-associated GBS. Red text indicates the number of records and, where applicable, the number of drugs.

The JADER dataset used in this study contained 4,707,221 records in the Drug table, 1,568,295 records in the Reaction table, and 949,124 records in the Demographic table (Figure 1). After merging these three tables, the analysis dataset consisted of 2,794,953 records, among which 1873 reports (0.067%) involved drug-associated GBS.

### 2.2. Drugs Showing Disproportionality Signals for GBS and Patient Characteristics

**Figure 2:**
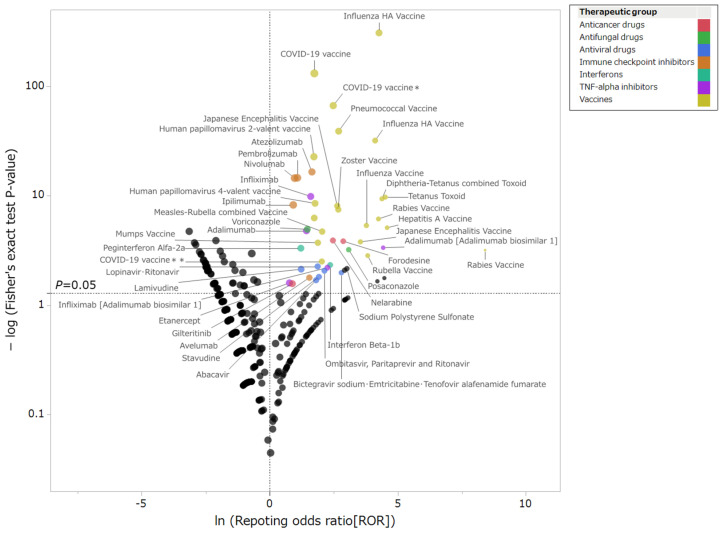
Relationships between drugs and GBS. In this scattergram volcano plot, the *x*-axis represents lnROR, and the *y*-axis represents −log_10_ (*p*-value). The dotted horizontal line indicates a *p*-value of 0.05 based on Fisher’s exact test. The color of each point represents the therapeutic class of the drug. Drugs showing signals for GBS are located in the upper right area of the plot. The asterisks (* and **) indicate COVID-19 vaccine reports that shared the same generic name but could not be distinguished as separate medicinal products in JADER.

Among more than 4000 drugs that could be analyzed in JADER, 45 drugs were identified as potentially associated with GBS (Supplementary Table S1). By therapeutic class, these agents included 19 vaccines, 5 immune checkpoint inhibitors, 4 tumor necrosis factor α (TNF-α) inhibitors, 6 antiviral drugs, 3 anticancer drugs other than immune checkpoint inhibitors, 2 antifungal drugs, and 2 interferons. The breakdown of reported cases by drug category was as follows: vaccines, 927 reports (49.5%); immune checkpoint inhibitors, 268 reports (14.3%); TNF-α inhibitors, 54 reports (2.9%); antiviral drugs, 22 reports (1.2%); anticancer drugs other than immune checkpoint inhibitors, 17 reports (0.9%); antifungal drugs, 17 reports (0.9%); and interferons, 15 reports (0.8%). The disproportionality results for drugs and GBS are plotted in Figure 2.

The number of reports by vaccine type totaled 354 for coronavirus disease 2019 (COVID-19) vaccines, 98 for COVID-19 vaccines *, 250 for influenza HA vaccines, 23 for influenza HA vaccines (A/H1N1), 56 for human papillomavirus 2-valent vaccines, 19 for human papillomavirus 4-valent vaccines, 50 for pneumococcal vaccines, 14 for hepatitis B vaccines, 10 for Japanese encephalitis vaccines, nine for zoster vaccines, eight for measles-rubella combined vaccines, seven for mumps vaccines, six for diphtheria-tetanus combined toxoid, and six for tetanus toxoid. The JADER dataset did not include detailed information on COVID-19 vaccine types or influenza vaccine strains. An asterisk (*) was used only for COVID-19 vaccines when reports shared the same generic name but could not be distinguished as different medicinal products. No reports of GBS were identified for respiratory syncytial virus (RSV) vaccines in this study.

The number of reports by immune checkpoint inhibitor was as follows: 91 for nivolumab, 76 for pembrolizumab, 54 for ipilimumab, 43 for atezolizumab, and 4 for avelumab. Durvalumab, cemiplimab, and tremelimumab did not show disproportionality signals for drug-associated GBS.

The numbers of reports for TNF-α inhibitors were 26 for infliximab, 14 for adalimumab, 11 for etanercept, and 3 for infliximab (biosimilar 1).

The numbers of reports for antiviral drugs were 7 for lamivudine, 4 for lopinavir/ritonavir, 3 for ombitasvir/paritaprevir/ritonavir, 3 for stavudine, 3 for abacavir, and 2 for bictegravir/emtricitabine/tenofovir alafenamide fumarate.

Other drugs showing disproportionality signals for GBS included the anticancer drugs gilteritinib, nelarabine, and forodesine; the antifungal agents voriconazole and posaconazole; and the interferon preparations pegylated interferon alpha-2a and interferon beta-1b.

The characteristics of patients in reports of drug-associated GBS are presented in Table 1. Notably, reports of GBS associated with human papillomavirus 2-valent and 4-valent vaccines and the mumps vaccine were exclusively in female patients and were concentrated in younger individuals. In contrast, reports associated with tetanus toxoid involved only male patients, whereas most other drugs and vaccines were reported predominantly in adults.

### 2.3. Time-to-Event Analysis and Weibull Distribution

**Figure 3:**
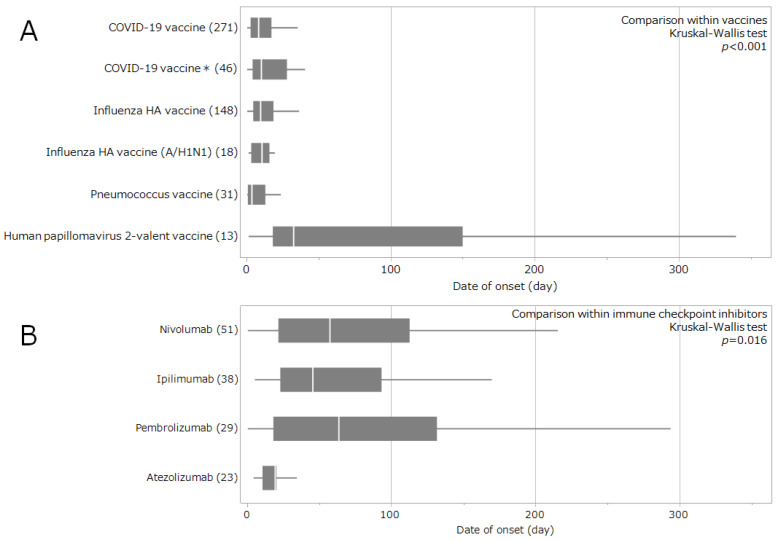
Time to onset of GBS associated with vaccines and immune checkpoint inhibitors. (**A**) Comparison among vaccines. (**B**) Comparison among immune checkpoint inhibitors. For drugs administered multiple times, the time to onset was defined as the interval from treatment initiation (first dose) to onset. The numbers in parentheses indicate the number of reported cases for each drug. Box-and-whisker plots show the time to onset. The center line indicates the median; the box represents the interquartile range (IQR, 25th–75th percentiles); and the whiskers extend to 1.5 × IQR or to the minimum and maximum values if no data fall beyond this range. The asterisk (*) indicates COVID-19 vaccine reports that shared the same generic name but could not be distinguished as separate medicinal products in JADER.

The time to onset of drug-associated GBS for vaccines and immune checkpoint inhibitors, together with the corresponding Weibull parameters, is presented in Table 2 and Figure 3. For vaccines, the median time to GBS onset (range) was 8.5 days (0.5–225.5) for COVID-19 vaccines, 10.5 days (0.5–347.5) for COVID-19 vaccines *, 10.5 days (0.5–212.5) for influenza HA vaccines, 10.5 days (1.5–51.5) for influenza HA vaccines (A/H1N1 strain), 4.5 days (0.5–212.5) for pneumococcal vaccines, and 32.5 days (1.5–352.5) for human papillomavirus 2-valent vaccines (Table 2). For COVID-19 vaccines, COVID-19 * vaccines, influenza HA vaccines, and pneumococcal vaccines, the Weibull shape parameter (β) indicated an early-failure pattern (β < 1, with the upper bound of the 95% confidence interval < 1), suggesting a higher reporting frequency early after administration followed by a decline over time (Table 2). A significant difference in time to onset was observed among vaccines (*p* < 0.001; Figure 3A).

For the immune checkpoint inhibitors nivolumab, ipilimumab, pembrolizumab, and atezolizumab, the median times to GBS onset (range) were 57.5 days (0.5–359.5), 45.5 days (5.5–169.5), 63.5 days (0.5–314.5), and 19.5 days (4.5–147.5), respectively. For many immune checkpoint inhibitors, the Weibull shape parameter (β) approximated a random-failure pattern (approximately β = 1), suggesting that the reporting frequency did not show a clear temporal change (Table 2). A significant difference in time to GBS onset was observed among immune checkpoint inhibitors (*p* = 0.016; Figure 3B).

As presented in Table 3, the median (range) time to GBS onset for COVID-19 vaccines * was 16.5 days (1.5–287.5) for men and 4.5 days (0.5–347.5) for women (*p* = 0.005). For influenza HA vaccines (A/H1N1 strain), the median time to GBS onset was significantly longer in men than in women (14.5 days [2.5–51.5] vs. 5.5 days [1.5–16.5], *p* = 0.032). No significant differences were observed for the other vaccines or immune checkpoint inhibitors. As shown in Table 4, no significant age-related differences in time to GBS onset were observed for vaccines, and immune checkpoint inhibitors were reported only in patients aged 20 years or older.

## 3. Discussion

### 3.1. Drugs Showing Disproportionality Signals for GBS

Vaccines effectively prevent infectious diseases because pathogen-derived antigens are presented by antigen-presenting cells, which activate helper T cells and stimulate B cells, thereby inducing antibody production and immunological memory, enabling a rapid and powerful immune response in the event of reinfection [18]. Several vaccines have been reported to be associated with a risk of GBS, and this study also suggested possible associations. In this study, COVID-19 and influenza vaccines accounted for the majority of reports of vaccine-associated GBS.

This study identified disproportionality signals for GBS associated with “COVID-19 vaccine” and “COVID-19 vaccine *” (Figure 2). A meta-analysis reported an increased risk of GBS after adenovirus-vector COVID-19 vaccination [19]. Patone et al. reported a twofold increased risk of GBS within 28 days after adenovirus-vector COVID-19 vaccination (IRR = 2.04), whereas mRNA-based vaccines were not associated with an increased risk [20]. By contrast, SARS-CoV-2 infection itself increased the risk of GBS approximately fivefold (IRR = 5.25) [20]. Our findings are broadly consistent with previous reports, although direct comparisons among COVID-19 vaccine types were not possible because JADER does not provide sufficiently detailed product information.

This study identified disproportionality signals for GBS associated with “Influenza HA vaccine” and “Influenza HA vaccine (A/H1N1)” (Figure 2). Influenza vaccination has been reported to carry a relative risk of 1.41 for GBS [21]. The 1976 swine influenza vaccine was thought to induce anti-ganglioside antibodies, potentially contributing to an increased risk of GBS [22]. Our findings are broadly consistent with previous reports. However, comparisons among influenza vaccine products or strains were not possible because those details were unavailable in JADER.

The present study also identified potential disproportionality signals for GBS associated with vaccines other than COVID-19 and influenza vaccines (Figure 2). Reports of GBS associated with pneumococcal vaccine [23], human papillomavirus vaccine [24], Japanese encephalitis vaccine [25], zoster vaccine [26], measles-rubella combined vaccine [27], and diphtheria-tetanus combined toxoid vaccines [28] have been described previously, and signals were also identified for these vaccines in the present study. Using U.S. Vaccine Adverse Event Reporting System data (1990–2005), Souayah et al. identified few GBS reports across vaccines and no disproportionality signals [29]. Recent real-world evidence has suggested a possible association between RSV vaccines and GBS in older adults [30]. However, no reports of GBS associated with RSV vaccines were identified in this study. Epidemiological evidence regarding the risk of GBS associated with vaccines other than COVID-19 and influenza vaccines remains limited; therefore, further investigation is warranted [31].

In the present study, disproportionality signals for GBS were identified for the immune checkpoint inhibitors nivolumab, pembrolizumab, ipilimumab, atezolizumab, and avelumab (Figure 2). Immune checkpoint inhibitors exert their antitumor effects by inhibiting the cytotoxic T lymphocyte-associated protein 4 (CTLA-4) pathway, which suppresses the initial stage of T-cell activation, and the programmed death-1 (PD-1)/programmed death-ligand 1 (PD-L1) pathway, which tumor cells use for immune evasion [32,33]. In addition to their expression in malignant tumors, PD-1/PD-L1 receptors are also expressed in activated immune cells and peripheral tissues [34]. Immune checkpoint inhibitors are therefore known to cause immune-related adverse events (irAEs), including T-cell- and antibody-mediated GBS [35,36]. The incidence of serious neurological adverse events associated with immune checkpoint inhibitors has been reported to be lower than 1% [37]. Nivolumab [38], pembrolizumab [39], ipilimumab [40], atezolizumab [41], and avelumab [42] have been reported to be potentially associated with the risk of GBS. In contrast, no disproportionality signals for GBS were detected for some immune checkpoint inhibitors, including the PD-L1 inhibitor durvalumab and the CTLA-4 inhibitor tremelimumab. A study based on the World Health Organization (WHO) VigiBase reported that no disproportionality signals for GBS were detected in association with PD-L1 inhibitors; however, a potential signal could not be excluded [43]. Like PD-1 inhibitors, PD-L1 inhibitors inhibit the binding of PD-L1 to PD-1, which reduces inhibitory signals to T cells, thereby promoting the activation of tumor-specific T cells and suppressing tumor growth [44]. As the mechanisms of irAEs are believed to be similar between PD-1 and PD-L1 inhibitors, future research is needed to determine differences in adverse-event profiles among PD-1, PD-L1, and CTLA-4 inhibitors [45].

In the present study, disproportionality signals for GBS were identified for the TNF-α inhibitors infliximab, adalimumab, etanercept, and infliximab (biosimilar 1) (Figure 2). TNF-α inhibitors are widely used to treat autoimmune diseases, such as rheumatoid arthritis and inflammatory bowel disease [46]. Adverse events reported in association with TNF-α inhibitors include demyelination [47] and forms of peripheral neuropathy, including GBS [48]. Adalimumab [49], infliximab [50], and etanercept [51] have been reported to be potentially associated with the risk of GBS. Hypothesized mechanisms include immune dysfunction attributable to multifunctional TNF-α inhibition [52] and autoantibody induction [53]; however, these mechanisms have not been fully elucidated [54]. The present study also identified a disproportionality signal for GBS associated with an infliximab biosimilar. Although there are insufficient data on the risk of GBS associated with TNF-α inhibitor biosimilars, the present findings suggest that, if the reference product carries a risk of GBS, similar precautions may also be warranted for its biosimilars.

In this study, we identified disproportionality signals suggesting a potential association between six antiviral drugs and GBS (Figure 2). Although the Japanese package inserts for antiviral drugs include a warning about GBS, no specific reports have directly linked these drugs to GBS. Restoration of immune function following antiviral therapy may be associated with the development of GBS; however, this may reflect an autoimmune response triggered by the virus itself or treatment-related immune reconstitution inflammatory syndrome rather than a direct effect of the drugs [55,56].

This study identified disproportionality signals for GBS associated with anticancer drugs other than immune checkpoint inhibitors, as well as antifungal drugs and interferon preparations (Figure 2). Although there have been reports of associations between nelarabine [57], voriconazole [58], posaconazole [59], and peginterferon alfa-2a [60] and GBS, further investigation is needed because the available evidence remains limited.

Disproportionality analysis is based on the number of spontaneously reported adverse event cases and does not account for detailed clinical information, such as disease course, risk factors, or alternative causes. For example, Guillain–Barré syndrome is often triggered by viral infections, which may confound the associations observed in spontaneous reporting databases. Therefore, the signals identified in this study should be interpreted as hypothesis-generating rather than as evidence of a causal relationship.

### 3.2. Timing of Vaccine- and Immune Checkpoint Inhibitor-Associated GBS Onset

In this study, the most common time to onset for vaccine-associated GBS was 1–3 weeks for COVID-19, influenza, and pneumococcal vaccines and 1–3 months for the human papillomavirus 2-valent vaccine, although the reporting frequency tended to decrease thereafter (Table 2, Figure 3A). A systematic review of GBS cases associated with COVID-19 vaccines reported that more than 80% of cases occurred within 3 weeks of vaccination [61]. An analysis using the U.S. Vaccine Adverse Event Reporting System reported that the median time to GBS onset following COVID-19 vaccination was 10 days, the median patient age was 55 years, and half of the patients were male [62]. In this study, the type of COVID-19 vaccine administered was unknown, and there was a slight female predominance. However, the median time to GBS onset was 8.5 days for the COVID-19 vaccine and 10.5 days for the COVID-19 vaccine *, consistent with previous reports (Table 2). Regarding the relationship between influenza vaccination and GBS, a prior report found that GBS cases were concentrated within 5 weeks of vaccination [63]. Analyses of seasonal influenza vaccines identified a peak incidence rate 2–4 weeks after vaccination [64,65], consistent with the median time to onset of 10.5 days for influenza vaccines in the present study (Table 2). Furthermore, women tended to develop symptoms earlier than men after COVID-19 and influenza vaccination (Table 3). A previous report suggested that women may develop earlier and more severe clinical symptoms of GBS than men after COVID-19 vaccination [66]. There have also been reports of GBS occurring within 2–4 weeks after pneumococcal vaccine administration [67,68], and a similar pattern was suggested in the present study (Table 2, Figure 3A). However, GBS most commonly emerged 1–3 months after human papillomavirus 2-valent vaccine administration in the present study, representing a later onset than that observed for other vaccines (Table 2, Figure 3A). Previous studies did not identify a link between human papillomavirus vaccination and GBS [69]. Because the human papillomavirus vaccine is intended primarily for the prevention of cervical cancer, its target population is young women. In this study, most patients with human papillomavirus vaccine-associated GBS were girls in their teens, which differed from the patient backgrounds observed for other vaccines (Table 3 and Table 4). This difference in time to onset likely reflects the target population of the vaccine and should be considered when interpreting the observed associations. Children have immature immune systems, including immature regulatory T cells and Th17 cells, and myelination is incomplete; the impact of demyelination may therefore differ from that in adults [70]. The incidence of GBS in children (0.62 cases/100,000 person-years) is lower than that in adults (2.66 cases/100,000 person-years) [71]. In addition, women appear to have a lower risk of developing GBS than men [72]. The human papillomavirus vaccine is also administered in multiple doses over a defined period. These differences could explain the observed later onset of GBS following human papillomavirus vaccination in this study.

In this study, the most common time to onset of drug-associated GBS was 1–3 months for nivolumab, ipilimumab, and pembrolizumab and 2–4 weeks for atezolizumab (Table 2, Figure 3B). Repeated administration of immune checkpoint inhibitors leads to continuous inhibition of the PD-1/PD-L1 and CTLA-4 pathways, which is believed to promote the expansion of autoreactive T cells and the production of anti-neuronal antibodies, resulting in an autoimmune response targeting peripheral nerves [73,74]. Previous reports have indicated that GBS associated with immune checkpoint inhibitors occurs approximately 5.4 [75] and 8.2 weeks [76] after treatment initiation, in line with the current findings for the PD-1 inhibitors nivolumab and pembrolizumab and the CTLA-4 inhibitor ipilimumab. In contrast, the time to GBS onset for the PD-L1 inhibitor atezolizumab was earlier in the present study, at 19.5 days (Table 2, Figure 3B). There are no published studies comparing the timing of drug-associated GBS onset among immune checkpoint inhibitors. However, PD-L1 inhibitors, such as atezolizumab, cause fewer irAEs than PD-1 inhibitors [77,78]. Although both PD-L1 and PD-1 inhibitors block the PD-1/PD-L1 pathway, PD-L1 inhibitors block the B7-1/PD-L1 pathway while sparing the PD-1/PD-L2 pathway, unlike PD-1 inhibitors [79,80,81]. The findings of this study suggest possible differences in the mechanisms underlying GBS associated with PD-1 and PD-L1 inhibitors, potentially involving the PD-1/PD-L2 or B7-1/PD-L1 pathways; however, these observations are hypothesis-generating. The impact of these pathways on immune function in drug-associated GBS warrants further investigation.

### 3.3. Limitations

Spontaneous adverse event reporting databases, such as JADER, are subject to inherent biases and limitations that should be considered when interpreting the results.

First, reporting bias may occur due to increased awareness of GBS following regulatory warnings or published reports, potentially leading to stimulated reporting for specific drugs. In addition, such databases rely on voluntary reports, and the total number of exposed patients is unknown, making direct risk estimation impossible [82]. Serious and well-recognized adverse events tend to be overreported, whereas mild or unknown events may be underreported [83,84]. When multiple drugs are administered, it is often difficult to identify the causative agent [85]. Furthermore, data quality issues should be considered, as JADER contains missing values and potential data entry errors; therefore, data cleaning was performed as thoroughly as possible. However, detailed analyses were limited by the lack of information on COVID-19 vaccine types and influenza vaccine strains.

Second, because this study employed disproportionality analysis for signal detection, the findings should be interpreted as exploratory, and false-positive signals cannot be excluded [86]. Disproportionality analysis is based on the number of reports rather than detailed clinical content of individual cases, and therefore does not allow for direct risk estimation or causal inference. In the signal evaluation of this study, signals were assessed using a combination of the ROR, Fisher’s exact test *p*-value, and a predefined threshold for the number of reports; however, because direct risk estimation and causal inference were not possible, careful interpretation is warranted.

Third, misclassification may have occurred because GBS diagnoses were not clinically validated at the individual case level and were based on reported terms. In addition, the use of standardized MedDRA queries (SMQs) may have introduced heterogeneity in case identification. The SMQ for GBS includes a broad range of Preferred Terms, some of which may represent conditions not directly related to drug exposure, such as autoimmune disorders (e.g., Bickerstaff’s encephalitis) or infection-associated syndromes (e.g., Zika virus-associated GBS). Furthermore, confounding factors, such as underlying diseases, concomitant medications, and infections (e.g., preceding viral or bacterial infections known to trigger GBS), could not be fully accounted for in this analysis. These factors may have influenced the observed disproportionality signals.

Fourth, the time-to-onset analysis may be subject to reporting bias, particularly for events with longer latency [87,88]. Reporting patterns may differ between products with well-established associations with GBS and those without, potentially affecting the observed distributions [89]. In addition, the Weibull distribution analysis should be interpreted with caution, as it is based on spontaneous reporting data and may be influenced by incomplete or biased reporting [87,89].

Finally, disproportionality analysis is based on the number of reports rather than detailed clinical content of individual cases. Because this study did not include a detailed clinical evaluation of individual cases, it was not possible to assess temporal relationships, alternative etiologies, or the strength of causality between drugs and GBS. Therefore, the identified signals should be interpreted with caution and further evaluated through detailed clinical review and well-designed epidemiological studies to validate these findings.

## 4. Materials and Methods

### 4.1. Detection of Disproportionality Signals for GBS

#### 4.1.1. Construction of the JADER Analysis Data Table

This study analyzed data registered in the JADER database from April 2004 to February 2025 [16]. The JADER Drug (e.g., drug name, drug involvement, start date, end date), Reaction (e.g., adverse events, onset date), and Demographic tables (e.g., basic patient information, such as gender, age, weight) were used in this study. Drugs in the Drug table were assigned to three categories based on their involvement in adverse events: suspected drugs, concomitant drugs, and drug interactions. Only the suspected drug data were used in this study. Furthermore, each drug was assigned an Anatomical Therapeutic Chemical Classification System (ATC) code for drug classification [90]. Adverse events in the Reaction table were based on the ICH International Medical Dictionary for Regulatory Activities (MedDRA)/Japanese version 27.1 [91]. Adverse events in the Reaction table can be grouped into specific medical conditions using SMQs [92]. The SMQ was applied as a broad case-finding tool for signal detection, allowing for the identification of potentially relevant cases. In this study, drug-associated GBS was defined using the nine GBS and related disease preferred terms within GBS (SMQ code: 20000131, narrow scope): acute motor axonal neuropathy, acute motor–sensory axonal neuropathy, Bickerstaff’s encephalitis, chronic inflammatory demyelinating polyradiculoneuropathy, demyelinating polyneuropathy, GBS, Miller Fisher syndrome, subacute inflammatory demyelinating polyneuropathy, and Zika virus-associated GBS. Not all Preferred Terms included in the SMQ necessarily represent confirmed GBS, and the identified cases may include heterogeneous conditions, such as autoimmune or infection-related disorders. In this study, the term “drug-associated GBS” refers to GBS cases identified from spontaneous reports and does not imply a causal relationship between drugs and GBS. A column was added to the Reaction table to indicate the presence or absence of drug-associated GBS. Overlapping cases between the Drug and Reaction tables were eliminated using the method reported by Hirooka et al. [93,94]. In the Demographic table, age was treated as a continuous variable. Specifically, ages were converted to 105 years for those in their 100s, 95 years for those in their 90s, 85 years for those in their 80s, 75 years for those in their 70s, 65 years for those in their 60s, 55 years for those in their 50s, 45 years for those in their 40s, 35 years for those in their 30s, 25 years for those in their 20s, 15 years for those in their 10s, and 5 years for those under 10. The three tables were joined using identification numbers (IDs) to create a data table for JADER analysis (Figure 1).

#### 4.1.2. Drugs Showing Disproportionate Reporting of Drug-Associated GBS and Patient Characteristics

**Figure 4:**
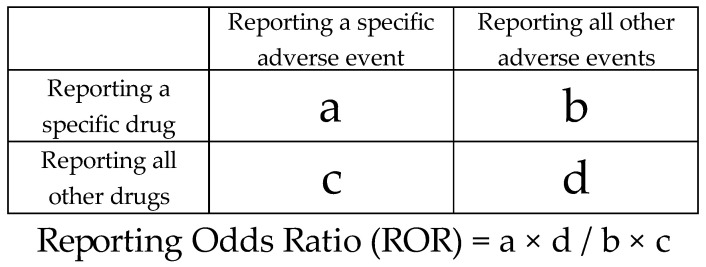
Cross-tabulation and formula used to calculate the ROR for an adverse event. The cross-tabulation is structured with reports for the suspected drug, all other reports, reports with an adverse event, and reports without an adverse event (a–d indicate the number of reports).

In this study, all drugs available in the JADER analysis data table were evaluated. A 2 × 2 contingency table was constructed for each drug in relation to drug-associated GBS (Figure 4). Using each 2 × 2 contingency table, three indices were calculated and evaluated: the ROR, Fisher’s exact test *p*-value, and the total number of reports for each drug (Figure 4). The 2 × 2 contingency table was corrected by adding 0.5 to all cells (Haldane–Anscombe 1/2 correction) to avoid instability in estimates when any cell was zero [95,96]. The ROR is an important indicator of signal detection in disproportionality analysis of adverse events and safety in pharmacovigilance [97]. The ROR is widely used in pharmacoepidemiologic studies because of its high sensitivity and low bias [98]. However, traditional signal detection indices, such as the ROR, may overestimate signals and produce unstable statistical estimates when reports are infrequent [99]. To address this issue, the EudraVigilance guidelines recommend a minimum number of reports to ensure a stable signal [100]. To prevent overlooking commonly used drugs, we set the total number of reports for each drug at 100 as a threshold (a + b > 100 in Figure 4) [101]. Furthermore, we used Fisher’s exact test to assess the independence of drugs and GBS in the 2 × 2 contingency table (Figure 4). Therefore, in this study, a drug was considered to show a signal when it met all of the following criteria for disproportionate reporting of drug-associated GBS: ROR > 1, Fisher’s exact test *p*-value < 0.05, and ≥100 reports [102].

To visually interpret the associations of approximately 4000 drugs with GBS, we created a scatter plot (volcano plot) of the ROR and *p*-value calculated using Fisher’s exact test. This volcano plot presents lnROR on the *x*-axis and −log_10_ (*p*-value) on the *y*-axis [103,104]. This scatter plot is equivalent to the volcano plot frequently used in bioinformatics to understand trends in gene expression.

We also investigated the characteristics of patients with reported drug-associated GBS, including gender and age.

Due to the nature of data from spontaneous reporting systems, this study relied on quantitative signal detection using disproportionality analysis without adjustment for confounding factors or clinical validation of cases.

### 4.2. Onset Timing of Drug-Associated GBS

#### 4.2.1. Construction of a Data Table for Time-to-Onset Analysis

We performed a time-to-event analysis of GBS reports in the JADER database for vaccines and immune checkpoint inhibitors and classified the onset patterns using the Weibull distribution [105,106]. From the Drug table, we used “identification number (ID)”, “drug involvement”, “drug name”, and “administration start date”. Regarding drug involvement, only suspected drugs were used. Administration start dates were converted to 8-digit YYYYMMDD format. Twelve-digit dates were truncated to the first eight digits, 8-digit dates were retained, 6-digit dates were converted by assigning the 15th day of the month, and 4-digit dates were treated as missing. We then extracted records with valid 8-digit administration start dates between 1 January 1960 and 28 February 2025. If a drug ID had multiple administration dates, the earliest was considered the administration start date. The Reaction table was used to extract reports of drug-associated GBS. The onset dates of drug-associated GBS in the Reaction table were cleaned to eight digits (yyyymmdd) using the same procedure applied to the Drug table. If a patient (same ID) had multiple dates of GBS onset, the earliest onset date was used. The Drug and Reaction tables were linked using IDs. The time to onset was calculated as the difference between the onset date and the administration start date after conversion to date format, plus 0.5 days as a continuity correction. Only values between 0.5 and 365.5 days were included in the analysis to reduce the influence of outliers, such as implausible values due to data entry errors or very long intervals for which causal interpretation is difficult, and to mitigate potential reporting bias because time to onset may be reported differently depending on prior knowledge of drug-GBS associations. For drugs administered multiple times, we examined the interval from treatment initiation (first dose) to the date of GBS onset.

#### 4.2.2. Evaluation of Adverse Event Onset Profiles

The timing of onset of drug-associated GBS for vaccines and immune checkpoint inhibitors was evaluated, and the onset patterns were classified using the Weibull distribution [107]. In the Weibull distribution, the scale parameter (α) represents the dispersion of the distribution, and the shape parameter (β) characterizes the failure pattern. A β value < 1 indicates early failure, with a higher hazard shortly after exposure that decreases over time; β = 1 indicates random failure with a constant hazard; and β > 1 indicates wear-out failure, with an increasing hazard over time [108]. In this study, the time to onset of drug-associated GBS was analyzed using the Weibull distribution to evaluate onset patterns.

### 4.3. Statistical Analysis

All analyses were performed using JMP Pro 18.0.0 (SAS Institute Inc., Cary, NC, USA), and *p* < 0.05 was considered statistically significant.

## 5. Conclusions

This study identified disproportionality signals for GBS associated with several drugs and vaccines using the JADER database. Disproportionality signals for drug-associated GBS were detected for vaccines, including COVID-19 and influenza vaccines; immune checkpoint inhibitors, including nivolumab and pembrolizumab; TNF-α inhibitors; antivirals; anticancer drugs other than immune checkpoint inhibitors; antifungal agents; and interferons, suggesting possible involvement of drugs that affect immune function. Regarding time-to-onset patterns, GBS reports were most frequently observed within 1–3 weeks after administration of COVID-19, influenza, and pneumococcal vaccines and within 1–3 months after administration of human papillomavirus 2-valent vaccines, with reporting frequency decreasing thereafter. Among immune checkpoint inhibitors, the most frequently reported time to onset was 1–3 months for nivolumab, ipilimumab, and pembrolizumab and 1–3 weeks for atezolizumab; however, no clear temporal trend in reporting was observed. The signals identified in this study through disproportionality analysis should be interpreted cautiously, as they may reflect reporting biases and other limitations inherent in spontaneous reporting systems and do not constitute evidence of causal relationships. These findings are primarily intended for hypothesis generation and early signal detection. Further pharmacoepidemiological and clinical studies are required to validate these findings.

## Figures and Tables

**Table 1:** Characteristics of patients involved in reports of drug-associated GBS.

Drug	N	Gender Male/Female/Unknown and Not Reported	Age Median (Min–Max)
Vaccines			
COVID-19 vaccine	354	165/185/4	55 (5–95)
COVID-19 vaccine *	98	56/42/0	45 (15–85)
Influenza HA vaccine	250	131/115/4	55 (5–85)
Influenza HA vaccine (A/H1N1)	23	13/10/0	65 (5–75)
Human papillomavirus 2-valent vaccine	56	0/56/0	15 (15–35)
Human papillomavirus 4-valent vaccine	19	0/19/0	15 (15–45)
Pneumococcal vaccine	50	28/15/7	75 (45–85)
Hepatitis B vaccine	14	7/7/0	25 (5–35)
Japanese encephalitis vaccine	10	5/5/0	10 (5–15)
Zoster vaccine	9	4/5/0	65 (55–75)
Measles-rubella combined vaccine	8	7/1/0	15 (5–55)
Mumps vaccine	7	0/7/0	15 (5–15)
Diphtheria-tetanus combined toxoid	6	2/4/0	15 (15–15)
Tetanus toxoid	6	6/0/0	30 (25–45)
Immune checkpoint inhibitors			
Nivolumab	91	62/26/3	65 (35–85)
Pembrolizumab	76	44/27/5	75 (25–85)
Ipilimumab	54	37/17/0	65 (45–85)
Atezolizumab	43	26/14/3	75 (45–95)
Avelumab	4	3/0/1	75 (75–75)
Others			
Infliximab	26	22/4/0	65 (15–75)

The asterisk (*) indicates COVID-19 vaccine reports that shared the same generic name but could not be distinguished as separate medicinal products in JADER.

**Table 2:** Onset status and Weibull distribution of drug-associated GBS.

Drug	Time to Onset (Days)	Weibull Distribution			N
	Median (Min–Max)	Scale Parameter α (95% CI)	Shape Parameter β (95% CI)	Failure Pattern	
Vaccines					
COVID-19 vaccine	8.5 (0.5–225.5)	13.41 (11.21–16.00)	0.71 (0.65–0.77)	Early failure type	271
COVID-19 vaccine *	10.5 (0.5–347.5)	27.43 (16.27–45.28)	0.61 (0.49–0.75)	Early failure type	46
Influenza HA vaccine	10.5 (0.5–212.5)	15.44 (12.66–18.76)	0.87 (0.77–0.98)	Early failure type	148
Influenza HA vaccine (A/H1N1)	10.5 (1.5–51.5)	13.36 (8.23–21.12)	1.09 (0.74–1.51)	Random failure type	18
Pneumococcal vaccine	4.5 (0.5–212.5)	10.83 (5.44–20.94)	0.57 (0.43–0.72)	Early failure type	31
Human papillomavirus 2-valent vaccine	32.5 (1.5–352.5)	81.92 (35.55–179.17)	0.77 (0.48–1.13)	Random failure type	13
Immune checkpoint inhibitors					
Nivolumab	57.5 (0.5–359.5)	83.75 (62.50–110.90)	1.03 (0.82–1.27)	Random failure type	51
Ipilimumab	45.5 (5.5–169.5)	65.52 (50.42–84.10)	1.35 (1.03–1.71)	Wear-out failure type	38
Pembrolizumab	63.5 (0.5–314.5)	92.13 (57.51–144.19)	0.86 (0.63–1.14)	Random failure type	29
Atezolizumab	19.5 (4.5–147.5)	33.68 (21.36–51.90)	1.02 (0.74–1.33)	Random failure type	23

The asterisk (*) indicates COVID-19 vaccine reports that shared the same generic name but could not be distinguished as separate medicinal products in JADER.

**Table 3:** Time to onset and Weibull distribution of drug-associated GBS by gender.

Drug	Gender	Time to Onset (Days)	Weibull Distribution		N	*p* Value ^#^
		Median (Min–Max)	Scale Parameter α (95% CI)	Shape Parameter β (95% CI)		
Vaccines						
COVID-19 vaccine						0.432
	Male	8.5 (0.5–175.5)	13.64 (10.77–17.18)	0.81 (0.71–0.92)	122	
	Female	8 (0.5–242.5)	13.44 (10.28–17.48)	0.66 (0.58–0.74)	144	
COVID-19 vaccine *						0.005
	Male	16.5 (1.5–287.5)	40.01 (22.62–68.82)	0.70 (0.53–0.90)	30	
	Female	4.5 (0.5–347.5)	11.49 (4.04–31.15)	0.53 (0.37–0.72)	16	
Influenza HA vaccine						0.416
	Male	9.5 (0.5–71.5)	13.71 (10.74–17.36)	0.98 (0.83–1.15)	79	
	Female	10 (0.5–108.5)	16.20 (12.11–21.46)	0.89 (0.74–1.06)	68	
Influenza HA vaccine (A/H1N1)						0.032
	Male	14.5 (2.5–51.5)	19.65 (11.20–33.32)	1.33 (0.77–2.03)	10	
	Female	5.5 (1.5–16.5)	7.03 (3.57–13.24)	1.28 (0.68–2.11)	8	
Human papillomavirus 2-valent vaccine						—
	Male	—	—	—	—	
	Female	32.5 (1.5–352.5)	81.92 (35.55–179.17)	0.77 (0.48–1.13)	13	
Pneumococcal vaccine						0.496
	Male	3 (0.5–23.5)	6.07 (3.09–11.39)	0.80 (0.53–1.12)	18	
	Female	4.5 (0.5–31.5)	8.23 (3.98–16.23)	0.98 (0.58–1.47)	11	
Immune checkpoint inhibitors						
Nivolumab						0.422
	Male	57.5 (0.5–359.5)	76.83 (53.49–108.62)	1.01 (0.77–1.29)	35	
	Female	67 (7.5–215.5)	99.97 (59.12–163.25)	1.09 (0.71–1.59)	16	
Ipilimumab						0.649
	Male	46.5 (5.5–112.5)	59.91 (45.95–76.97)	1.64 (1.17–2.20)	26	
	Female	44.5 (7.5–169.5)	77.04 (42.21–135.01)	1.13 (0.68–1.71)	12	
Pembrolizumab						0.853
	Male	63.5 (1.5–314.5)	104.55 (55.05–191.02)	0.89 (0.57–1.28)	16	
	Female	97 (0.5–293.5)	81.32 (35.13–179.83)	0.85 (0.49–1.33)	11	
Atezolizumab						0.203
	Male	20.5 (4.5–147.5)	46.27 (23.87–85.81)	0.97 (0.61–1.43)	13	
	Female	15.5 (8.5–20.5)	16.97 (14.36–19.85)	4.85 (2.63–7.91)	9	

^#^ Wilcoxon/Kruskal–Wallis test. The asterisk (*) indicates COVID-19 vaccine reports that shared the same generic name but could not be distinguished as separate medicinal products in JADER.

**Table 4:** Time to onset and Weibull distribution of drug-associated GBS by age.

Drug	Age	Time to Onset (Days)	Weibull Distribution		N	*p* Value ^#^
		Median (Min–Max)	Scale Parameter α (95% CI)	Shape Parameter β (95% CI)		
Vaccines						
COVID-19 vaccine						0.610
	<20 y	9.5 (0.5–153.5)	14.96 (7.54–28.65)	0.75 (0.52–1.00)	19	
	≥20 y	7.5 (0.5–242.5)	13.41 (11.12–16.12)	0.71 (0.65–0.77)	250	
COVID-19 vaccine *						0.524
	<20 y	347.5 (347.5–347.5)	—	—	1	
	≥20 y	9.5 (0.5–287.5)	24.57 (14.81–39.98)	0.64 (0.51–0.78)	45	
Influenza HA vaccine						0.311
	<20 y	12.5 (1.5–108.5)	18.52 (12.42–27.18)	0.98 (0.75–1.23)	31	
	≥20 y	9.5 (0.5–90.5)	13.94 (11.27–17.15)	0.92 (0.80–1.06)	116	
Influenza HA vaccine (A/H1N1)						0.333
	<20 y	16.5 (16.5–16.5)	—	—	1	
	≥20 y	10.5 (1.5–51.5)	12.98 (7.73–21.16)	1.06 (0.71–1.47)	17	
Human papillomavirus 2-valent vaccine						0.894
	<20 y	32.5 (1.5–352.5)	82.62 (33.25–193.62)	0.74 (0.45–1.11)	12	
	≥20 y	52.5 (52.5–52.5)	—	—	1	
Pneumococcal vaccine						—
	<20 y	—	—	—	0	
	≥20 y	3.5 (0.5–31.5)	6.86 (4.25–10.79)	0.86 (0.63–1.12)	29	
Immune checkpoint inhibitors						
Nivolumab						—
	<20 y	—	—	—	0	
	≥20 y	57.5 (0.5–359.5)	83.75 (62.50–110.90)	1.03 (0.82–1.27)	51	
Ipilimumab						—
	<20 y	—	—	—	0	
	≥20 y	45.5 (5.5–169.5)	65.52 (50.42–84.10)	1.35 (1.03–1.71)	38	
Pembrolizumab						—
	<20 y	—	—	—	0	
	≥20 y	63.5 (0.5–314.5)	90.05 (55.02–143.74)	0.84 (0.61–1.12)	28	
Atezolizumab						—
	<20 y	—	—	—	0	
	≥20 y	19.5 (4.5–147.5)	33.68 (21.36–51.90)	1.02 (0.74–1.33)	23	

^#^ Wilcoxon/Kruskal–Wallis test. The asterisk (*) indicates COVID-19 vaccine reports that shared the same generic name but could not be distinguished as separate medicinal products in JADER.

## Data Availability

The data presented in this study are openly available from the JADER (Japanese Adverse Drug Event Report) database on the PMDA website (accessed on 16 March 2025).

## Supplementary Materials

The following supporting information can be downloaded at: https://www.mdpi.com/article/10.3390/ph19050688/s1, Table S1: Drugs associated with disproportionality signals for Guillain–Barré syndrome (GBS).

## Abbreviations

The following abbreviations are used in this manuscript:

ATC	Anatomical Therapeutic Chemical Classification System
COVID-19 vaccine	Coronavirus disease 2019 vaccine
CTLA-4	Cytotoxic T lymphocyte-associated protein 4
GBS	Guillain–Barré syndrome
ID	Identification number
irAEs	Immune-related adverse events
JADER	Japanese Adverse Drug Event Report
MedDRA/J	Medical Dictionary for Regulatory Activities/Japanese version
PD-1	Programmed death-1
PD-L1	Programmed death-ligand 1
PMDA	Pharmaceuticals and Medical Devices Agency
ROR	Reporting odds ratio
RSV	Respiratory syncytial virus
SMQ	Standardized MedDRA Query
TNF-α	Tumor necrosis factor alpha
WHO	World Health Organization
